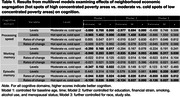# Neighborhood economic segregation and cognitive decline among midlife women: Study of Women’s Health Across the Nation

**DOI:** 10.1002/alz.086157

**Published:** 2025-01-09

**Authors:** Jinshil Hyun, Carol A. Derby, Bradley M. Appelhans, Emma Barinas‐Mitchell, Rebecca C Thurston, Carrie A. Karvonen‐Gutierrez, Monique Hedderson, Imke Janssen, Mary D. Schiff

**Affiliations:** ^1^ Albert Einstein College of Medicine, Bronx, NY USA; ^2^ Department of Neurology, and Department of Epidemiology and Population Health, Albert Einstein College of Medicine, Bronx, NY USA; ^3^ Rush University, Chicago, IL USA; ^4^ University of Pittsburgh, Pittsburgh, PA USA; ^5^ Department of Psychiatry, School of Medicine, University of Pittsburgh, Pittsburgh, PA USA; ^6^ University of Michigan, Ann Arbor, MI USA; ^7^ Kaiser Permanente, Oakland, CA USA; ^8^ UPMC Children’s Hospital, Pittsburgh, PA USA

## Abstract

**Background:**

Neighborhood context includes conditions of the environment where people spend their time (e.g., work, play, seek health care) and it may affect residents’ cognitive health. Prior studies have documented that lower neighborhood‐level socioeconomic status (SES) is associated with cognitive impairment and with risk for Alzheimer’s disease and related dementia (ADRD). However, most prior studies assessed neighborhood SES within administrative boundaries (e.g., census tract) without considering the distribution of SES among adjacent neighborhood areas (i.e., spatial segregation). The present study aims to investigate whether spatial segregation of neighborhood SES is associated with longitudinal cognitive decline among women over midlife.

**Method:**

In the Study of Women’s Health Across the Nation (SWAN), we examined effects of neighborhood economic segregation on levels and rates of cognitive decline in processing speed, working memory, and verbal episodic memory (N = 941; age at cognitive baseline 49‐60 years; mean follow‐up 9.1±4.3 years; 46% non‐Hispanic White, 24% Black, 12% Chinese, 18% Japanese). To assess neighborhood economic segregation, we used census tract poverty data (i.e., 150% below the federal poverty limit) based on participant’s residential address and local spatial statistics and identified hot spots (high concentrated poverty areas) and cold spots (low concentrated poverty areas). Covariates were baseline age, race/ethnicity, education, study site, and time‐varying financial strain, smoking, alcohol use, and menopause status.

**Result:**

Women living in hot spots showed worse working memory at baseline and more rapid decline in episodic memory over time relative to those living in cold spots (Table 1). The significant effects persisted after controlling for absolute neighborhood poverty level within each census tract.

**Conclusion:**

Midlife women residing in economically segregated areas experienced more rapid decline in episodic memory over an average of 9 years. Living in more socioeconomically segregated areas may impact individuals’ health‐related behaviors, use of medical facilities, psychosocial states, and availability of community social capital, which are all important risk/protective factors for cognitive impairment and ADRD. Future studies need to consider spatial associations among neighborhood areas to better characterize neighborhood environments influencing individuals’ cognitive health and should investigate individual and contextual mechanisms that underlie the observed associations.